# Attitudes of female carriers of X-linked hypohidrotic ectodermal dysplasia towards prenatal treatment and their decisions during a pregnancy with a male fetus

**DOI:** 10.1186/s13023-025-03710-7

**Published:** 2025-04-15

**Authors:** Holm Schneider, Michael Schneider, Massimiliano Lia, Dorothy K. Grange, Smail Hadj-Rabia, Angus Clarke, Mary Fete, Agnes Jaulent, Marlene Guiraud, Anthony Odibo, Florian Faschingbauer

**Affiliations:** 1 Center for Ectodermal Dysplasias, University Hospital Erlangen, Loschgestr. 15, 91054 Erlangen, Germany; 2 Department of Obstetrics, University Hospital Leipzig, Leipzig, Germany; 3 Division of Genetics and Genomic Medicine, Department of Pediatrics, Washington University, St. Louis, USA; 4 Department of Dermatology, Reference Centre for Genodermatoses and Rare Skin Diseases, Hopital Necker-Enfants Malades, University of Paris-Cité, Paris, France; 5 Institute of Medical Genetics, Cardiff University School of Medicine, Cardiff, Wales, U.K.; 6 National Foundation for Ectodermal Dysplasias, Fairview Heights, USA; 7 EspeRare Foundation, Geneva, Switzerland; 8 Pierre Fabre Medicament, Lavaur, France; 9 Department of Obstetrics and Gynecology, Washington University, St. Louis, USA

**Keywords:** Ectodermal dysplasia, Tooth germ, Ultrasound, Prenatal therapy, Protein replacement

## Abstract

**Background:**

X-linked hypohidrotic ectodermal dysplasia (XLHED) is a severe genetic disorder that may be treatable with short-term protein replacement therapy during fetal development. This is currently being investigated in a multicenter clinical trial. Affected fetuses can be identified by the number of tooth germs during a routine ultrasound scan in mid-gestation. To understand the attitudes of female XLHED carriers towards prenatal treatment and ultrasonographic screening of the fetus, we analyzed an earlier and a very recent survey among those women and the actual decisions of potential trial participants.

**Methods:**

Initial analyses were based on a self-administered survey of 167 female XLHED carriers conducted in 2011. A similar questionnaire was completed 12 years later by 72 female XLHED carriers aged 18–45 years. Subsequently, both the path to diagnosis and further decision-making of the first 33 pregnant women screened for participation in the EDELIFE trial were investigated.

**Results:**

Most women diagnosed with XLHED considered this disease as an obstacle to having children: About one third had decided not to have children, another third would monitor their pregnancy using invasive genetic testing. In both surveys, a small number of women stated that they would consider termination of pregnancy depending on the test result. When it came to participating in the clinical trial, 80% were likely to take part (17% moderately likely, 63% very likely). Among the first pregnant women screened for this trial, 48% underwent invasive tests, while 52% relied on non-invasive tooth germ imaging for fetal XLHED diagnosis. One pregnancy with an affected fetus was terminated, another one resulted in a miscarriage, one woman declined to participate in the trial, and 12 women (80%) decided to have the affected fetuses treated.

**Conclusion:**

Ultrasound-based screening and prenatal treatment of the fetus are viewed positively by the vast majority of female XLHED carriers.

**What’s already known about this topic?**

- XLHED is caused by a genetic deficiency of the signaling protein ectodysplasin A1, resulting in a congenital lack of ectodermal derivatives, such as hair, teeth, and sweat glands.
- Ultrasonographic assessment of fetal tooth buds during mid-trimester organ screening allows the detection of male fetuses who may be candidates for prenatal therapy with a recombinant replacement protein, which is still an experimental approach.

**What does this study add?**

- The study provides novel data on the attitudes and perceptions of female carriers of an X-linked congenital disease. Although being a carrier of XLHED may impact the decision to have children, ultrasound-based testing and treatment of affected male fetuses seems to be the preferred reproductive choice of women who are carriers and wish to become pregnant.
- With other fetal therapies in development, this could be the beginning of a paradigm shift in prenatal counseling.

## Introduction

X-linked hypohidrotic ectodermal dysplasia (XLHED) is a rare genetic disorder affecting approximately 4 per 100,000 live male births [1]. It is caused by a deficiency of the signaling protein ectodysplasin A1 (EDA1) during fetal development [2, 3], resulting in a partial or complete absence of sweat glands and perspiration, paucity of other skin appendages derived from the embryonic ectoderm, and missing teeth [4]. As sweating is essential for human thermoregulation, especially during intense physical activities, febrile illnesses, or in hot environments, the congenital lack of sweat glands leads to lifelong problems, including dangerous episodes of overheating [5, 6]. We are conducting a pivotal clinical trial to treat affected boys *in utero* with an EDA1 replacement protein (EDELIFE trial) [7]. If successful, this treatment could change the lives of individuals with EDA1 deficiency by giving them the capacity to sweat, regulate their body temperature and develop more teeth. The first prenatal administrations of the therapeutic protein (on a named-patient basis) took place in 2016 [8]; normal ability to perspire was achieved and has persisted for eight years in the two oldest subjects. The absence of circulating EDA1 proved that these children would not have been able to sweat if they had been left untreated [9].

The replacement protein must be delivered within the tight developmental window for sweat gland formation in order to permanently correct the main clinical problem associated with XLHED. Fortunately, the insufficient formation of teeth in all affected boys allows a non-invasive prenatal diagnosis as early as five months before birth by counting the fetal tooth buds during a routine ultrasound examination [10]– just in time for protein replacement therapy. Tooth germ sonography proved to be highly specific and reliable in the prenatal detection of XLHED [11].

A fetal therapy approach such as the one described above might not be readily accepted [12]. Familial, medical, social, cultural, and economic factors must be taken into account so that the family can make an informed decision. It is therefore worth finding out what women of childbearing age think about fetal therapy. Gathering stakeholder views can provide the basis for discussions with regulatory authorities on a planned or current fetal therapy trial and help to address the priorities and needs of affected families in future research. The objective of this study was to understand the attitudes of female XLHED carriers towards the novel treatment method and the ultrasound-based testing of the fetus, which may change over the years. We have thus compared the respective results of a previous and a very recent survey among carrier women with or without affected children and the decisions of the first potential trial participants. We hypothesized that the majority of pregnant women eligible for the ongoing EDELIFE trial would actually participate.

## Methods

### Historical survey

The XLHED Carrier Outlook toward Reproduction Survey (X-CORS), a cross-sectional, observational study based on a self-administered survey (Clinicaltrials.gov identifier: NCT01398813) was designed by Edimer Pharmaceuticals, Inc., in order to improve the understanding of the decisions that female XLHED carriers make regarding their reproduction, genetic testing, and potential treatments of the congenital condition. The survey consisted of closed questions, restricting participants to one of a limited set of possible answers. After ethical approval from the institutional review board of Maine Medical Partners Pediatric Specialty Care Portland (USA) had been obtained, the survey was made available under https://www.surveymonkey.com/s/X-CORS. Responses were collected between August 2nd and October 11th, 2011. A total of 167 women from the USA (*n* = 129) or various other countries (*n* = 38) who were either genetically confirmed carriers of XLHED or had two or more cardinal signs of this disorder together with a family history indicating X-linked inheritance and were at least 18 years old took part in this study. The data were reviewed and analyzed using descriptive statistics.

### EDELIFE trial and second survey in 2023

The EDELIFE trial (ClinicalTrials.gov identifier: NCT04980638) is a prospective, open-label, genotype-match controlled, pivotal multicenter clinical trial to investigate the efficacy and safety of intra-amniotic ER004 administration as a prenatal treatment for male subjects with XLHED [7], that was started in November 2021. ER004 has received Breakthrough Therapy and Orphan Drug Designation in the United States and benefits from the PRIME (Priority Medicines) program of the European Medicines Agency and from Orphan Drug Designation in Europe. Ethical approval was obtained from the institutional review boards of all participating university hospitals. The treatment consists of three ultrasound-guided intra-amniotic injections of ER004 two or three weeks apart, starting in gestational week 26. During each intervention, both amniotic fluid and maternal blood samples are collected for pharmacokinetic analyses.

An internet-based, self-administered survey comprising questions on the family history of XLHED, reproductive decisions, previous pregnancies, the clinical study awareness, and the intention to participate in the trial described above was designed by a multidisciplinary team of clinicians, patient representatives and experts from the market research company A + A who also programmed and hosted the respective website. A link to the survey was provided to women in the USA by the National Foundation for Ectodermal Dysplasias. This survey was completed online by 72 female XLHED carriers aged 18–45 years between July 14th and August 22nd, 2023.

### Statistical analysis

Data sets from the X-CORS and the second survey were analyzed using descriptive statistics and comparisons between the two surveys. A two-sided alpha of 0.05 was considered significant for all analyses.

### Prenatal ultrasonography

**Fig. 1:**
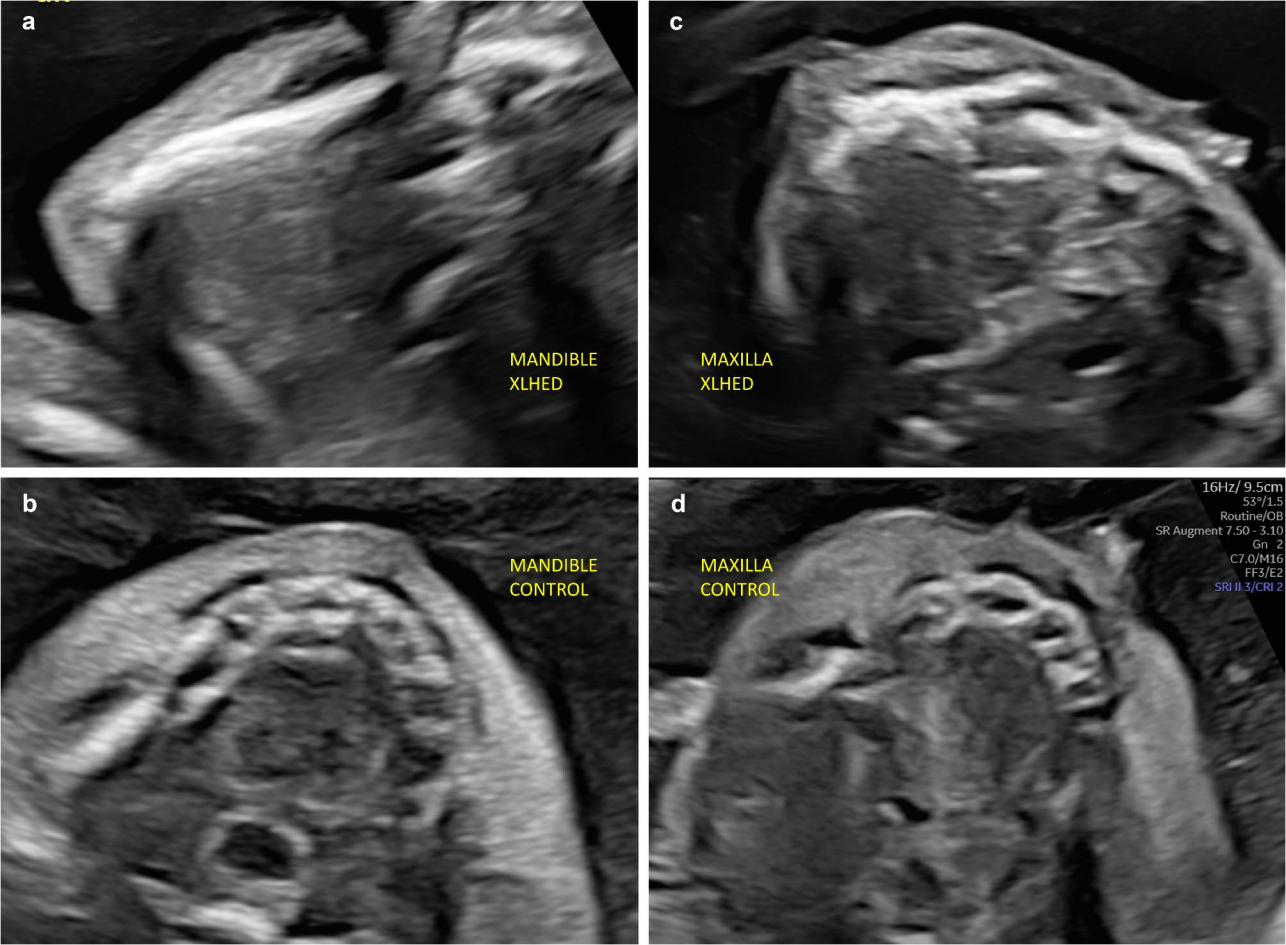
Transabdominal 2D scan of fetal tooth germs. Edentulous mandible (**a**) and only two tooth buds in the maxilla (**c**) of a 21-week old fetus with XLHED compared with mandible and maxilla of a healthy control fetus (**b, d**)

Pregnant women who participated in the EDELIFE trial underwent repeated ultrasound-based assessment of fetal tooth germ development. All ultrasonographers were experts in fetal medicine and had comparable levels of experience. Prior to the first screening for the study, they had received detailed instructions and had been trained using 2D-pictures and videos of tooth germ imaging. Ultrasound examinations were carried out at gestational ages of 20−24 weeks using standard high-end devices. The fetal maxilla and mandible were visualized and round hypoechogenic structures arranged in an arch-like fashion in the alveolar bone were identified as tooth germs. If the examiner counted fewer than six of them in either mandible or maxilla, their number was considered to be clearly reduced. In the case of unfavorable conditions for the examination, such as spine-up position of the fetus, tooth bud imaging was repeated at a later point in time. Figure 1 shows representative images of a fetus with XLHED (Figs. 1a, c) and the jaws of a healthy control fetus (Figs. 1b, d).

## Results

The XLHED Carrier Outlook toward Reproduction Survey (Survey #1) conducted in 2011 indicated that the majority of women carrying an *EDA* mutation saw XLHED as an obstacle to having children. Approximately two thirds of female disease carriers without children (64.9%) and more than half with affected sons or daughters stated that XLHED would impact their decision to become pregnant (Table 1). About one third had made a definitive decision not to have children. In 2.7–15.4% of women in the different subgroups, one or more previous pregnancies had been terminated due to XLHED. A similar proportion of survey participants said they would undergo invasive testing of the fetus in the event of pregnancy (Table 1) and consider an abortion depending on the result. Of note, if a potential prenatal therapy for affected boys were to be tested in a clinical trial, 85–94% of respondents stated that they would be moderately or highly likely to take part, with the highest likelihood among women with affected sons. There appeared to be no relevant differences between respondents from the USA (*n* = 129) and European women, mainly from France, Ireland and the UK (*n* = 23), or women from Australia (*n* = 8).

**Table 1:** Results of survey #1 among female XLHED carriers conducted in 2011

	Women without affected children (*n* = 37)	Women with affected sons (*n* = 117)	Women with affected daughters only (*n* = 13)
Stated that XLHED would impact their decision to have children	24 (64.9%)	62 (53.0%)	7 (53.8%)
One or more previous pregnancies terminated due to XLHED	1 (2.7%)	6 (5.1%)	2 (15.4%)
Stated that they would undergo invasive testing if pregnant and consider termination of pregnancy depending on the test result	4 (10.8%)	1 (0.9%)	2 (15.4%)
No statement on potential participation in a clinical trial of a prenatal therapy for affected boys	2 (5.4%)	2 (1.7%)	2 (15.4%)
Unlikely to participate in a clinical trial of a prenatal therapy for affected boys	2 (5.4%)	5 (4.3%)	0 (0%)
Moderately likely to participate	7 (18.9%)	16 (13.7%)	4 (30.8%)
Very likely to participate	26 (70.3%)	94 (80.3%)	7 (53.8%)

Twelve years later, after the start of the EDELIFE trial evaluating a prenatal therapy of XLHED, an online survey (Survey #2) was conducted in the USA, which confirmed some findings of Survey #1. It revealed that genetic counseling for women at risk of passing on XLHED was not always adequate, although the majority of them had received information about the likelihood of their children being affected, mostly given by geneticists or genetic counselors. Interestingly, fewer women indicated that XLHED would impact their decision to have children, and fewer had a history of terminations of pregnancy due to XLHED (Table 2). Only 20% of women without children and none of the mothers of affected sons or daughters stated that they would use pre-implantation genetic diagnosis or other techniques to select the embryos. Between 2.9% and 12.3% of respondents were willing to undergo invasive testing if pregnant and would consider termination of pregnancy depending on the test result (Table 2).

**Table 2:** Results of survey #2 among female XLHED carriers conducted in 2022, after the start of the EDELIFE trial

	Women without affected children (*n* = 30)	Women with affected sons (*n* = 34)	Women with affected daughters only (*n* = 8)
Stated that XLHED would impact their decision to have children	19 (63.3%)	11 (32.4%)	4 (50.0%)
One or more previous pregnancies terminated due to XLHED	1 (3.3%)	1 (2.9%)	0 (0%)
Stated that they would use pre-implantation genetic diagnosis or other techniques to select the embryos	6 (20.0%)	0 (0%)	0 (0%)
Stated that they would undergo invasive testing if pregnant and consider termination of pregnancy depending on the test result	1 (3.3%)	1 (2.9%)	1 (12.5%)
No statement on potential participation in the current clinical trial	5 (16.5%)	4 (11.8%)	0 (0%)
Unlikely to participate in the current clinical trial	6 (20.0%)	4 (11.8%)	2 (25.0%)
Moderately likely to participate	8 (26.7%)	2 (5.9%)	1 (12.5%)
Very likely to participate	11 (36.7%)	24 (70.6%)	5 (62.5%)

Five women without affected children (16.5%) and 4 women with affected sons (11.8%) did not respond to the question about their potential participation in the current clinical trial. Among the other women who made a statement about their willingness, 17% were moderately likely and 63% very likely to take part. The strongest affirmation came again from survey participants with affected sons (Table 2).

**Fig. 2:**
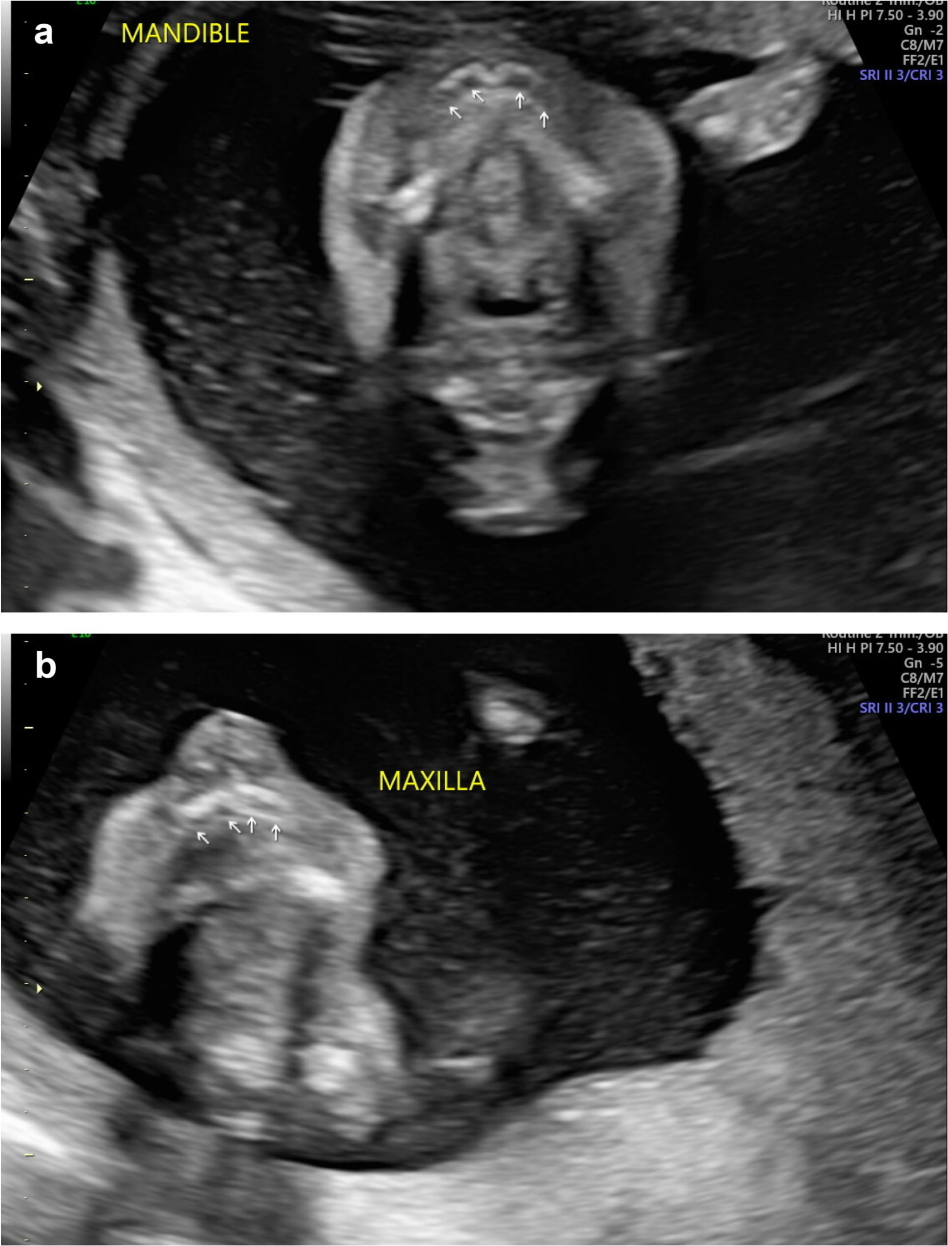
Unusual, ambiguous tooth germ images in a fetus with XLHED. Four anterior tooth buds in both mandible (**a**) and maxilla (**b**) of a 22-week old fetus, later confirmed to be affected by XLHED

Among 33 XLHED carriers pregnant with a male fetus who were screened for participation in the EDELIFE trial until May 2024, most women without affected children or with affected daughters relied on the non-invasive ultrasound-based diagnosis, whereas 60% of women with affected sons underwent invasive testing of the fetus (Table 3). Altogether, however, the majority (52%) preferred non-invasive ultrasonography for fetal XLHED diagnosis. In one of the women scheduled for tooth germ assessment, an ultrasound-based diagnosis was difficult to establish, because the fetal jaws were largely hidden in the maternal pelvis and tooth buds could not be visualized well enough. As the woman wanted to avoid invasive diagnostics, imaging of the tooth germs was repeated a week later. This time the ultrasound examination showed 4 anterior tooth buds in the mandible (Fig. 2a) and at least 5 tooth germs in the maxilla (Fig. 2b). The posterior parts of the jaws were not clearly visible. Therefore, the tooth germs could again not be counted completely. According to the EDELIFE study protocol, the diagnosis of XLHED can be made if fewer than 6 tooth buds are detected in either jaw. As neither mandible nor maxilla had been fully visualized, an amniocentesis was recommended to make a clear diagnosis, which proved that the fetus was affected. In all other cases, the ultrasonographic findings were later confirmed by genetic testing of amniotic fluid samples withdrawn in connection with the first intra-amniotic administration of the therapeutic protein.

**Table 3:** Actual decisions of the first 33 XLHED carriers pregnant with a male fetus who were screened for participation in the EDELIFE trial

	Women without affected children (*n* = 12)	Women with affected sons (*n* = 15)	Women with affected daughters only (*n* = 6)	Total number of subjects (*n* = 33)
Women who underwent invasive testing of the fetus	5 (41.7%)*	9 (60.0%)	2 (33.3%)	16 (48.5%)
Women relying on non-invasive ultrasound-based diagnosis (fetal tooth germ imaging)	7 (58.3%)	6 (40.0%)	4 (66.7%)	17 (51.5%)
Affected fetuses identified primarily by invasive testing	2/5	6/7	2/3	10/15
Affected fetuses identified primarily by ultrasonography	3/5	1/7	1/3	5/15
Pregnancy with an affected fetus terminated	0/5	1/7	0/3	1/15
Affected fetuses not enrolled in the trial due to miscarriage	1/5	0/7	0/3	1/15
Affected fetuses not enrolled in the trial because parents decided not to participate	0/5	1/7	0/3	1/15
Affected fetuses treated *in utero*	4/5	5/7**	3/3**	12/15

* including a pregnant woman who initially underwent ultrasound-based diagnostics and wanted to avoid invasive testing which was, however, strongly recommended to make a clear diagnosis

** including a total of three fetuses who were treated outside the clinical trial due to incompatible travel restrictions during the COVID-19 pandemic

A total of 15 affected fetuses had been identified by June 2024. One pregnancy was miscarried, another one was terminated, and one mother decided not to take part in the trial because she lived too far away from the study sites. Twelve of 15 fetuses were treated *in utero*, in three cases outside the clinical trial due to incompatible travel restrictions during the COVID-19 pandemic.

## Discussion

ER004, to our knowledge the first drug product being developed to treat a genetic disease *in utero*, seems to be highly appreciated by female carriers of XLHED. That is by no means a given but matches with the findings of other studies on different rare congenital disorders [12, 13]. According to Survey #1 more than a decade ago, XLHED had a strong impact on the decision to have children. In a more recent study by Leo et al. [14], 13 out of 50 women (26%) stated that prenatal detection of XLHED in the fetus would impact the continuation of pregnancy. In the last few years, however, knowledge has spread among affected families that the genetic deficiency of the signaling molecule EDA1 may be overcome before birth by minimally invasive administration of a replacement protein [15]. This is due in particular to the efforts of patient advocacy organizations to raise awareness of current clinical research. Since the EDELIFE trial began, with the possibility of having an affected son treated *in utero*, fewer women see XLHED as an obstacle to having children, fewer have had an abortion because of XLHED, and fewer are willing to undergo invasive testing if pregnant and consider termination of pregnancy depending on the test result. However, risks of bias and non-representative sample sizes in the data reported here and a study design that can only suggest such associations must be considered.

As neither the pregnant woman nor the fetus seem to develop antibodies against ER004 when administered intra-amniotically [16], treatment could even be possible in a subsequent pregnancy. This is due to the absence of relevant transplacental passage of the replacement protein from the fetus to the mother in the third trimester [8, 17], while anti-drug antibodies were detected after intravenous administration in non-pregnant females [16].

Tooth germ ultrasonography, sometimes combined with a screening for facial characteristics of ectodermal dysplasia, has been used in an increasing number of centers to detect XLHED in mid-gestation [11, 18–20]. Most XLHED carrier women appear to prefer non-invasive “testing” and do rely on it. Growing experience, however, does not rule out diagnostic uncertainties, as seen in this study. In rare cases, amniocentesis and genetic testing of fetal cells may be definitely required to make a clear diagnosis. Genotyping of the fetus is, of course, part of the study protocol for the EDELIFE trial, but can be done safely in connection with the first therapeutic intervention (e.g., withdrawal of amniotic fluid immediately before protein injection into the amniotic cavity). In our opinion, ultrasound-based tooth germ assessment could be the superior diagnostic tool for XLHED because it is readily performed in the mid-trimester fetal organ screening and consequently would recognize affected male fetuses even in women without any family history of the disease. If it were part of a routine fetal anatomy scan, the vast majority of fetuses in need of treatment could probably be detected prior to gestational week 25, provided that the ultrasound specialists are appropriately trained in recognizing fetal tooth buds.

However, the performance of tooth germ imaging as a screening test for the general population is unknown and would need to be assessed carefully before routine screening could be recommended for all pregnant women. In particular, the proportion of screen-positive results with a genetically confirmed diagnosis of XLHED (the positive predictive value of the test in the context of population screening) would be important to know, which may be very different from findings in the context of known carriers of XLHED. Both an acceptable positive predictive value and adequate training of ultrasound specialists would be essential requirements for such widespread screening.

A theoretical alternative, non-invasive prenatal testing of maternal blood (NIPT), is not yet available for XLHED. In general, this is more difficult to establish for an X-linked disorder as there is no affected paternal allele in the blood sample that would be genetically different from the maternal genome.

In the EDELIFE trial, fetal tooth bud counts in the screening visits are confirmed by additional magnetic resonance imaging prior to the first therapeutic intervention. Accuracy is important, because the number of erupted teeth or tooth germs at a later stage constitutes a secondary endpoint of the study. Once completed, the EDELIFE trial is expected to provide solid information both on the precision of prenatal tooth germ assessments and the efficacy of ER004 administrations in subjects with EDA1 deficiency. As the study highlights that ultrasound-based screening for a rare genetic disease can pave the way to successful ultrasound-guided treatment, it may also stimulate the clinical development of fetal therapies for other congenital disorders.

In conclusion, prenatal treatment is viewed positively by most female XLHED carriers. Gynecologists, obstetricians and genetic counselors are on the front line to inform affected families about this new option. XLHED can be diagnosed early enough during a routine ultrasound examination of the fetus by fetal tooth bud imaging. Despite the risk of bias and the small sample sizes of the cohorts investigated so far, this non-invasive and reliable diagnostic method seems to be the preferred procedure for XLHED carriers pregnant with a male fetus and is worthy of further exploration by specialists in prenatal medicine.

## Data Availability

The datasets used and analysed in this study are available from the corresponding author upon reasonable request.
